# A PCA-Based Approach for Very Early-Age Hydration Monitoring of Self-Compacting Concrete Using Embedded PZT Sensors

**DOI:** 10.3390/s23073627

**Published:** 2023-03-30

**Authors:** Qunfeng Liu, Yifan Mu, Xiaoting Li, Xing Wu, Xiang Ren

**Affiliations:** 1 School of Architecture and Civil Engineering, Xi’an University of Science and Technology, Xi’an 710054, China; 2 CCCC First Highway Consultants Co., Ltd., Xi’an 710068, China

**Keywords:** hydration monitoring, self-compacting concrete, principal component analysis, PZT, electromechanical impedance

## Abstract

This work proposed a novel approach based on principal component analyses (PCAs) to monitor the very early-age hydration of self-compacting concrete (SCC) with varying replacement ratios of fly ash (FA) to cement at 0%, 15%, 30%, 45%, and 60%, respectively. Based on the conductance signatures obtained from electromechanical impedance (EMI) tests, the effect of the FA content on the very early-age hydration of SCCs was indicated by the predominant resonance shifts, the statistical metrics, and the contribution ratios of principal components, quantitatively. Among the three, the PCA-based approach not only provided robust indices to predict the setting times with physical implications but also captured the liquid-solid transition elongation (1.5 h) during the hydration of SCC specimens with increasing FA replacement ratios from 0% to 45%. The results demonstrated that the PCA-based approach was more accurate and robust for quantitative hydration monitoring than the conventional penetration resistance test and the other two counterpart indices based on EMI tests.

## 1. Introduction

Self-compacting concrete (SCC) possesses better workability and flowability than ordinary Portland concrete due to the reduced friction between aggregates and cementitious materials caused by the addition of active minerals [1]. Fly ash (FA) is a class of environmentally friendly industrial waste that has promising potential as a mineral admixture to substitute cement. In the production of SCC with FA admixture, the early-age hydration of concrete is a complicated physiochemical process. The reaction between the cement, water, and FA determines the hydration rate and microstructure of concrete during curing, which further affects its strength gain and long-term performance in service. It has been reported that the variations in FA contents might retard or accelerate the setting process and final strength of SCC [2,3]. Thus, hydration monitoring is a crucial tool to bridge the mix proportion of concrete with its final physical property after curing.

In past decades, many conventional techniques, like the hydration heat method, Vicat needle test, and X-ray diffraction [2,4,5], have been proposed to monitor the hydration of cementitious materials. These techniques are inconvenient for in-situ hydration monitoring in concrete structures and are often used in laboratories. For hydration monitoring of concrete in the field, the wave propagation technique has been utilized to measure the early age hydration of SCC mixtures [6,7]. Zheng et al. [8] found that the setting times of SCC were highly dependent on their FA contents. However, this technique requires a pair of piezoelectric sensors with an intended wave propagation path that is vulnerable to environmental interferences in practical applications [9,10,11,12]. To address these issues, the electromechanical impedance (EMI) technique has been proposed by Soh and Bhalla [13] and validated by Shin et al. [14] using surface-bonded PZT patches for strength gain monitoring in concrete during curing ages from day 3 to day 28. Further, in order to monitor the very early-age hydration and setting processes, PZT sensors need to be embedded in concrete pastes from the very beginning of casting [15,16,17].

**Figure 1:**
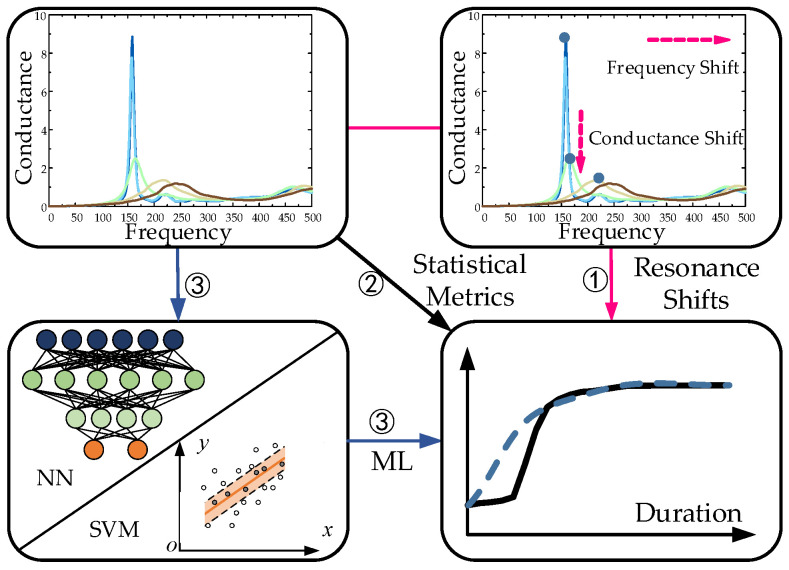
Flowchart of the EMI-based approaches for concrete strength gain prediction.

Based on the EMI signatures, the strength gain of concrete has been reported to be related to the predominant resonance shifts, statistical metrics, or indices obtained by machine learning [15,18]. Figure 1 shows the flowchart of the aforementioned approaches for predicting concrete strength gain based on the EMI technique. For example, empirical relationships between resonance shift and concrete strength were reported by Tawie et al. [19,20] via embedded PZT sensors. Furthermore, many statistical metrics have been verified to indicate the hydration process and even strength development quantitatively in concrete [20]. Furthermore, the electrical conductance shifts in concrete were validated to be related to the changes in the hydration process due to the supplementary cementitious materials and the water-to-binder (w/b) ratios [15,21]. Compared with the wave propagation technique, the EMI technique is less vulnerable to environmental interferences since it needs only one sensor and does not require a wave propagation path. However, the raw EMI signatures still contain intrinsic and extrinsic information induced by the concrete mix, fluctuation of the curing condition, installation error, etc. This would limit the accuracy and reliability of the EMI technique for hydration monitoring in concrete structures.

To address these limitations, machine learning (ML) methods can be utilized to process EMI data and thereby enhance the performance of the embedded PZT sensors [18]. Based on the measured EMI data, a gaussian support vector machine (SVM) model was utilized by Bansal et al. [22] to extract equivalent structural parameters of concrete and predict the strength gain during curing. A convolutional neural network (CNN) was trained by Ai et al. [23] to learn the raw EMI signatures for automated stress monitoring and damage identification in concrete. Furthermore, principal component analyses (PCAs) have been utilized to compress the measured conductance signatures before a further regression or classification was applied [24,25]. Among these ML algorithms, PCA has shown great potential in structural damage detection and health monitoring [26], since it can help eliminate the correlation among various variables and create new principal components to highlight the key features of the raw data. Thus, the PCA is expected to have the advantage of reducing non-principal information correlated in the EMI signatures. However, as far as we know, the research using PCA on the early hydration monitoring of concrete has not been reported yet.

The purpose of this work is to propose a PCA-based approach for quantitative hydration monitoring in SCC via embedded PZT sensors. The proposed approach will provide a robust indicator based on the raw EMI data and the contribution of the first principal component for hydration monitoring of SCC at very early ages. This indicator can not only capture the setting process of SCC but also reflect the effect of the FA replacement ratio. Compared with the approaches based on resonance shifts and statistical metrics, the PCA-based approach is more robust for hydration monitoring and more accurate in reflecting the effect of FA content on the settings of SCC.

## 2. Methodology

### 2.1. EMI Technique

Having direct and converse piezoelectric effects, PZT patches can be used as sensing and actuating transducers concurrently. By attaching a PZT patch to a host structure, the mechanical impedance of the host structure can be closely related to the electrical impedance of the PZT patch. When the PZT is excited by a sinusoidal voltage over a certain frequency range, the vibration in the PZT patch will excite the vibration of the host structure, which will be altered concurrently. Further, the electro-mechanical coupling effect of PZT will modulate the electric current across the PZT patch, which can be measured by an impedance analyzer and output as a complex electrical admittance. Liang et al. [27] first proposed a coupled electro-mechanical analysis of the one-degree-of-freedom (DOF) model to determine the structural responses of the host structure based on the EMI of the PZT patch integrated into a spring-mass-damper (SMD) system. Considering the electromechanical coupling effect between the PZT patch and the host structure, a simple model of the electromechanical admittance (EMA) can be derived from a one-dimensional system in axial vibration, as expressed in Equation (1):

(1) $$Y=G+Bj=j\omega \frac{bl}{h}\left[{\bar{\varepsilon }}_{33}^{T}-\frac{Z_{s}}{Z_{s}+Z_{a}}d_{31}^{2}{\bar{Y}}_{11}^{E}\right]$$

where *Y* is the electrical admittance, *G* is the electrical conductance (the real part of the admittance), *B* is the electrical susceptance (the imaginary part), *ω* is the angular frequency, and *j* is the imaginary unit. *Z_a_* and *Z_s_* are the mechanical impedances of the PZT patch and the attached host structure, respectively. *l*, *b*, and *h* represent the respective length, width, and thickness of the PZT patch. *d*_31_ and ${\bar{\varepsilon }}_{33}^{T}$ denote the piezoelectric coupling constant and complex dielectric constant at zero stress, respectively. ${\bar{Y}}_{11}^{E}$ is the complex Young’s modulus at zero electric fields. Based on Equation (1), any changes in the mechanical impedance of the host structures (*Z_s_*) can be reflected by the measured electromechanical admittances of PZT sensors.

**Figure 2:**
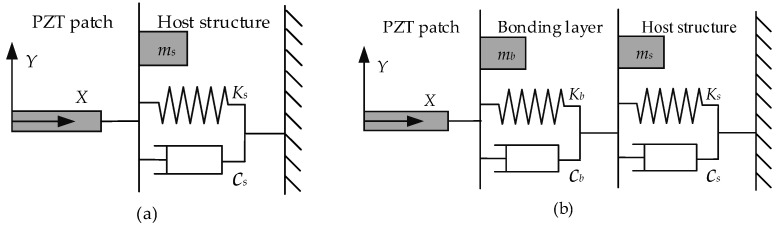
The one-DOF model (**a**) and the two-DOF model (**b**) for the embedded PZT sensors.

In practice, an embedded PZT sensor is usually packed with protective coatings. These coatings can be considered a bonding layer that will affect the dynamic coupling between the host structure and the embedded PZT patch. Thus, to obtain a more accurate prediction of host structure, the effect of the bonding layer on EMA signatures should be taken into account. A modified model was proposed by inserting an equivalent bonding layer, simplified as the additional SMD system, into the aforementioned one-dimensional model, thus obtaining the two-DOF model shown in Figure 2b. The electrical admittance can be expressed as [28]:

(2) $$Y=j\omega \frac{bl}{h}\left[{\bar{\varepsilon }}_{33}^{T}-d_{31}^{2}{\bar{Y}}_{11}^{E}+\frac{Z_{a}}{\xi Z_{s}+Z_{a}}d_{31}^{2}{\bar{Y}}_{11}^{E}\frac{\tan(kl)}{kl}\right]$$

(3) $$\xi =\frac{1}{1+K_{s}/K_{b}}$$

(4) $$Z_{a}=-j\frac{{\bar{Y}}_{11}^{E}bh}{\omega l}\frac{kl}{\tan(kl)}$$

(5) $$k^{2}=\rho \omega^{2}/{\bar{Y}}_{11}^{E}$$

where *Z_a_* is the PZT mechanical impedance; *ρ* is the density; ξ is a modification coefficient of the mechanical impedance of the host structure (*Z_s_*) and is determined by the dynamic stiffness ratio (*K_s_*/*K_b_*) of the host structure (*K_s_*) to the equivalent bonding layer (*K_b_*). From Equations (2) and (3), it can be concluded that the dynamic stiffness ratio (*K_s_*/*K_b_*) determines the modification coefficient of the mechanical impedance of the host structure and affects the measured electrical conductance. Thus, incorporating the effect of bonding layers will certainly alter the dynamic interaction between the PZT sensor and host structure [28]. In the two-DOF model, the stiffness (*K_b_*), damping (*c_b_*), and effective mass (*m_b_*) of the equivalent bonding layer can be considered explicitly. Although it is difficult to determine the exact *K_b_*, *c_b_*, and *m_b_* that represent the equivalent effect of the bonding layer, the modified two-DOF model can provide a more accurate description of the measured mechanical impedance.

### 2.2. Fabrication of the PZT-Based Sensors

**Figure 3:**
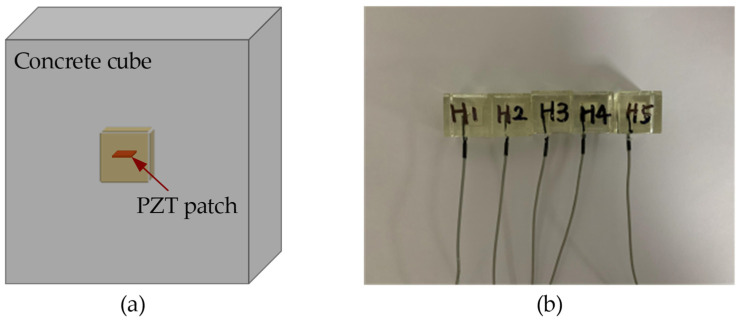
(**a**) Schematic layout of a cubic concrete specimen embedded with a PZT-based sensor; (**b**) fabricated PZT sensors packed with epoxy blocks.

PZT patches were usually packed with epoxy, cement, or marble blocks for protection against loading and environmental ingression under construction [29,30,31]. In this work, we fabricated the three types of PZT-based sensors at the same size for comparison. The fabrication of the sensors packed with cement and marble blocks followed the procedures suggested in previous studies [31,32]. For the easy position of PZT patches, the epoxy-packed PZT sensors were fabricated following the procedures: first, the bottom half of the cubic mold was cast with epoxy and half-hardened; then, a square PZT-5 patch with dimensions of 10 mm × 10 mm × 0.5 mm was positioned in the center of the cubic mold; finally, the top half of the mold was cast with epoxy and hardened altogether with the bottom half. To monitor the strength gain of concrete at a very early age, these PZT-based sensors need to be embedded into the concrete before casting and start monitoring from the very beginning of hydration. After packing, the fabricated PZT sensors were embedded into the concrete cubes for EMA measurement. The schematic layout of a typical cubic PZT sensor (dimensions: 20 mm × 20 mm × 20 mm) containing an embedded PZT patch (dimensions: 10 mm × 10 mm × 0.5 mm) is shown in Figure 3a. The fabricated PZT sensors used in this work are shown in Figure 3b.

Table 1 lists the electrical resistances and capacitances of the epoxy-packed PZT sensors (H1~H5) tested before and after packing. The electrical resistance of each PZT sensor was kept infinite after packing. The average capacitance of the PZT patches before packing was 2.3 nf and decreased by an average reduction of around 10.7% after being packed. A similar reduction in the electrical capacitances of the PZT patches was confirmed by previous experiments [29]. The small reduction in electrical capacity suggests that the influence of the epoxy coatings on the electromechanical coupling constant of the embedded PZT patches can be neglected.

**Figure 4:**
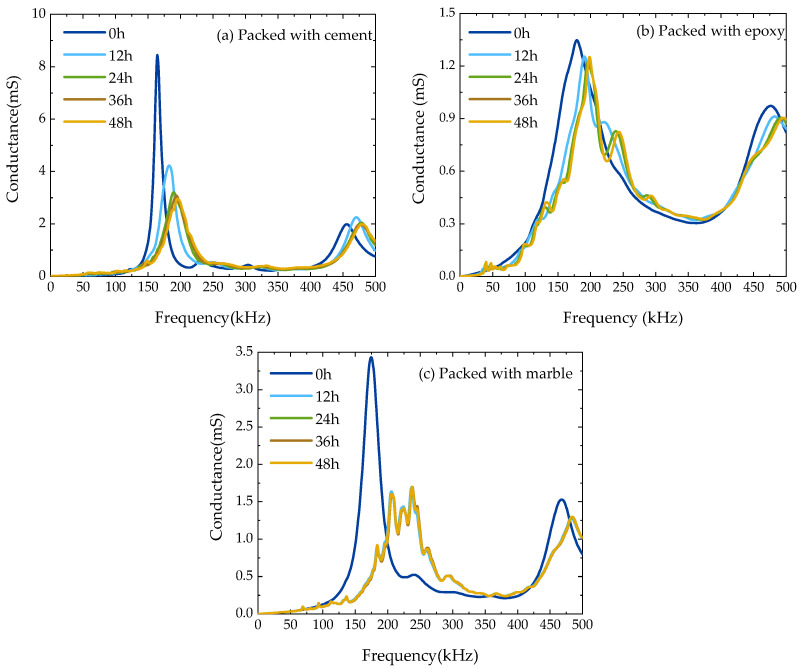
Conductance spectra measured from the PZT sensors with cement packing (**a**), epoxy packing (**b**), and marble packing (**c**) during fabrication.

Figure 4 shows the conductance signatures of the three types of PZT sensors measured during their fabrication processes. For each type of sensor, the change in conductance signatures is drastic at the first stages of curing and becomes negligible after a certain duration, which can be considered the curing completion time. The completion times of sensors packed in marble and epoxy blocks are 24 h and 36 h, respectively. Both are less than that of the PZT sensor packed in cement (over 48 h). It was also noted that the predominant resonance of the conductance signature continued to exist during the fabrication of the PZT sensors packed by either cement or epoxy, meaning a good resonance consistency between the PZT patch and its fabricated sensor. However, for the sensor packed in the marble block, the predominant resonance did not exist at the completion of its fabrication. Compared with the sensors packed with cement and marble, the epoxy-packed PZT sensors have advantages in stable electrical and electromechanical properties, quick fabrication, and good resonance consistency. Therefore, only the fabricated PZT sensors packed in epoxy blocks were used in the following studies.

## 3. Experimental Program

### 3.1. Preparation of SCC Specimens

Typical SCC specimens were cast into cubes (100 mm × 100 mm × 100 mm) following the mix proportion of a C40 ordinary Portland cement (OPC), consisting of water, cement at grade 42.5, river sands (fine aggregates), crushed stones with grain sizes ranging from 5 mm to 20 mm (coarse aggregates), limestone, water-reducing agents, and fly ashes. For each type of SCC specimen considered in this study, the water to binder (w/b) ratio remained constant at 0.37, and a specific portion of cement was replaced by the same mass of grade I fly ash (FA), thus obtaining SCCs with various FA replacement ratios by mass of binder, i.e., 0%, 15%, 30%, 45%, and 60% [33]. The considered mix proportions of the test SCC specimens with different FA replacement ratios are shown in Table 2, where the mix proportion of SCC-F30 specimens was taken as the reference with the expected compressive strength of 40 MPa.

Five sets of SCC specimens with different mix proportions were prepared using the same batch of materials. For each FA replacement ratio, ten specimens were prepared with a certain mix proportion, as listed in Table 2. Five of them were used for compressive tests and the other five for EMA tests. Before casting, a slump flow test was conducted for each fresh SCC mixture. Then, each specimen was cast in a cube mold with a fabricated PZT sensor pre-positioned in the center. After casting, each specimen was hardened in the mold for 24 h and then cured in water after demolding for the following 27 days. All specimens were cast and cured at the same temperature of 20 ± 2 °C to reduce experimental errors during curing.

### 3.2. Experimental Setups

SCC has good workability, which makes it easy to be self-compacted under gravity without any external vibrating efforts. Conventionally, both the slump flow test and penetrating resistance test were utilized to characterize the workability of the fresh SCC pastes at the beginning of hydration. For SCC mixtures with different FA replacement ratios (FA/binder) of 0%, 15%, 30%, 45%, and 60%, the slump expansion diameters are 312 mm, 400 mm, 585 mm, 630 mm, and 675 mm, respectively. This indicates that, at the same w/b ratio, the workability of SCC increases with the FA replacement ratio. As the replacement ratio increases beyond 30%, the slump expansion diameter is larger than 550 mm, which meets the requirement of the Chinese codes in terms of concrete self-compaction [33,34]. These results confirm the reported ball-bearing effect [35] induced by the large proportional fly ashes, leading to friction reduction among aggregates and thereby enhanced workability in concrete.

**Figure 5:**
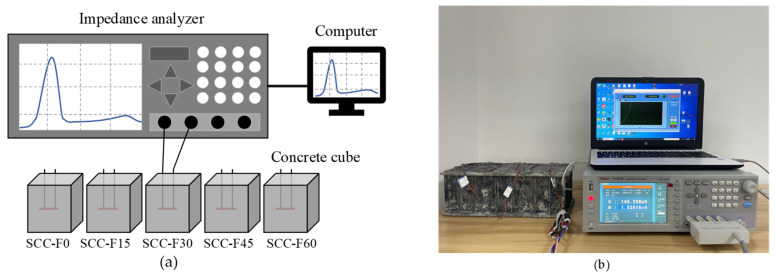
Schematic diagram (**a**) and equipment (**b**) of the electromechanical admittance testing system.

The schematic diagram of the experimental setup as well as the testing system used in this study are shown in Figure 5, where an impedance analyzer (TH2839, Changzhou Tonghui Electronic Co. Ltd., Changzhou, China) is adopted to measure the electrical admittance signals via the epoxy-packed PZT sensor embedded in the concrete specimens and to output the admittance signals to a computer for post-processing. The sinusoidal alternating voltage (with an amplitude of 1.0 V) was applied by the impedance analyzer to the embedded PZTs. An identical frequency sweeping range from 20 kHz to 500 kHz was selected for all specimens to standardize the measured EMI data and to identify the conductance changes easily. For each FA replacement ratio, five specimens were tested for electromechanical admittance. The electrical admittance signals contain both conductance (the real part) and susceptance (the imaginary part) signals. Since the susceptance signals are more susceptible to ambient temperature fluctuations, the conductance signals are usually used as signatures for monitoring the mechanical properties of the test specimens. For each specimen, the conductance signature was measured every hour from the very beginning of the casting up to 24 h under the same curing condition. The conductance signature measured at the first hour was taken as the baseline.

## 4. Results and Discussion

### 4.1. Hydration Monitoring Based on Penetration Resistance Tests

Based on the penetration resistance tests, the hydration process can be investigated by identifying the initial and final setting times of SCC specimens. According to the Chinese national standard (GB/T1346-2011) [36], the initial and final setting times can be defined as the instants when the penetration resistance value reaches 3.5 MPa and 28 MPa, respectively. Based on the penetration resistance tests, the initial and final setting times for the tested SCC specimens with different FA replacement ratios were obtained for comparison, as listed in Table 3. It is noted that both setting times increase with the increase in the FA replacement ratio. This demonstrates that the FA is less active than cement, which confirms the previous report that the SCC with a larger FA replacement ratio has a slower setting rate and delayed setting times [37]. However, the increasing trend in the gap duration between two settings with the FA replacement ratio cannot be observed in these penetration resistance tests. This contradicts the expectation that the hydration rate decreases with the increase of the FA replacement ratio [5], meaning that the penetration resistance test cannot accurately capture the duration elongation of the liquid-solid transition in the SCC mixture.

### 4.2. Hydration Monitoring Based on Predominant Resonance Shifts

**Figure 6:**
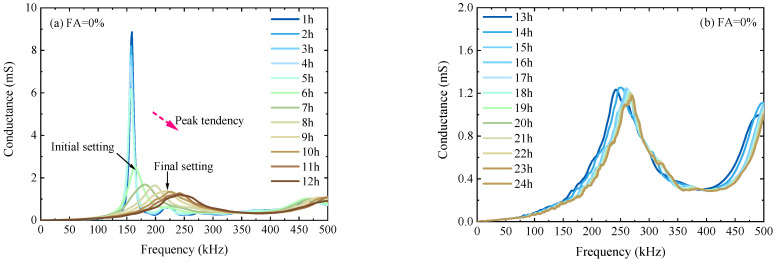

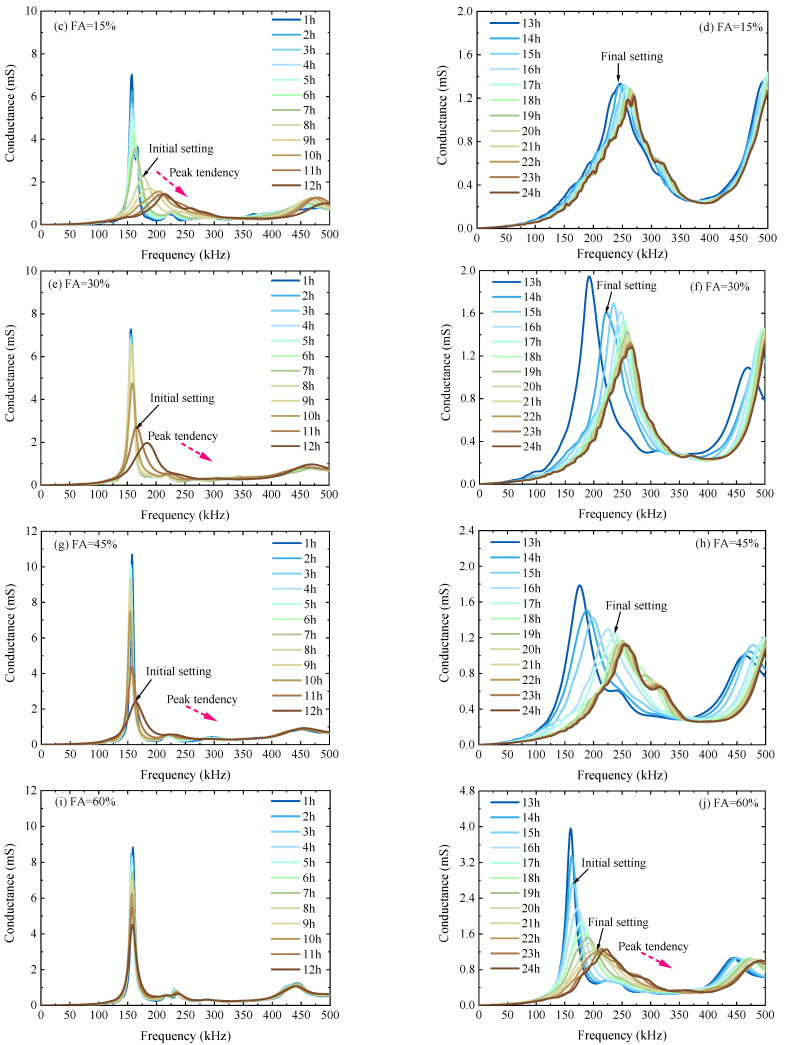
Conductance signatures for SCC specimens with different FA replacement ratios during curing from 1 h to 24 h.

Based on the electromechanical testing system, the electromechanical admittances of SCC specimens were recorded every hour after the initial casting for up to 24 h. Figure 6 shows the conductance signatures for the SCC specimens with five FA replacement ratios of 0%, 15%, 30%, 45%, and 60%, respectively. For each specimen with a specific FA replacement ratio, the conductance signatures measured from 1 h to 12 h (and from 13 h to 24 h) were presented separately for clarity over the same frequency range from 0 to 500 kHz.

As indicated in Figure 6, each conductance signature has a predominant peak, reflecting the resonance of the PZT sensor constrained by the SCC paste. With the increase in curing time, the predominant peak shifts downward and rightward in terms of frequency and amplitude, respectively. The resonance shifts with curing can be closely related to the changes in the mechanical impedances of host structures. Thus, the hydration process of SCC can be monitored by just tracking the amplitude shift or the frequency shift of the predominant resonance. In a liquid SCC mixture, the predominant peak approximately represents the resonance of a free PZT sensor. As the mixture transits from liquid to semi-solid, the amplitude of the predominant peak decreases to a certain value (~2.2 ms) that corresponds to the penetrating resistance at 3.5 MPa (initial setting). From this moment on, the rightward frequency shift of the resonance becomes drastic. Further, as the SCC paste reaches the solid state, the predominant resonance peak converges within the range from 240 kHz to 270 kHz with an amplitude of around 1.25 ms, which corresponds to the penetrating resistance at 28 MPa (final setting). From this point on, the downward amplitude shift of the predominant resonance becomes negligible.

**Figure 7:**
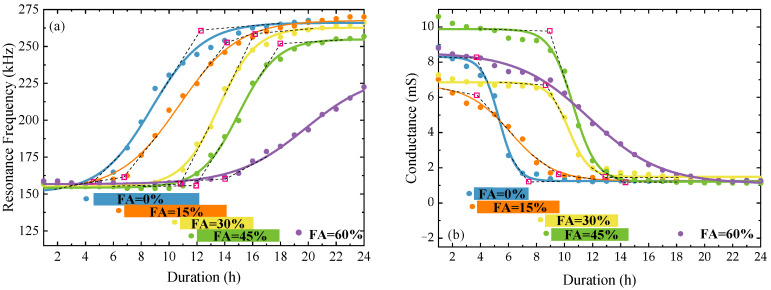
Resonance frequencies (**a**) and amplitudes (**b**) for SCC specimens with different FA replacement ratios during curing.

Figure 7a,b shows the changing curves of resonance frequency and amplitude with hydration duration for SCC specimens with different FA replacement ratios. For each case with a certain FA replacement ratio, the hydration process can be classified into three stages: the liquid stage, in which the resonance frequency and amplitude are nearly constant; the transition stage, in which the resonance frequency increases while the resonance amplitude decreases rapidly; and the solid stage, in which the resonance frequency and amplitude converge to be constant. Similar three-stage hydrations in concrete were also reported by Kong et al. [10] using the wave propagation approach via a pair of embedded PZT-based sensors. For each changing curve, the inflection between the first and second stages was fitted by using the Boltzmann sigmoid function to represent the initial setting, and the inflection between the second and third stages was fitted as the final setting. According to the fitted setting times, the transition duration of each case can be presented as a color bar and listed below. From the color bars, it can be observed that both the initial and final settings delay with the increase in FA replacement ratio. However, the elongation of the transition duration reported by experiments [8] cannot be captured by either resonance frequency shift or amplitude shift.

### 4.3. Quantitative Hydration Monitoring Based on the Statistical Indices

For quantitative monitoring of the mechanical impedances of concrete, statistical indices such as root mean square deviation (RMSD) and correlation coefficient (CC) have been proposed to quantify the changes in the conductance signatures [38]. These statistical indices stand on the assumption that most of the changes in the mechanical impedance of concrete during curing can be reflected by the changes in the measured conductance signatures over a certain frequency range. These indices can be expressed as:

(6) $$RMSD=\sqrt{\frac{\sum_{i}^{N}{\left(G_{i}^{1}-G_{i}^{0}\right)}^{2}}{\sum_{i}^{N}{\left(G_{i}^{0}\right)}^{2}}}$$

(7) $$CC=\frac{\frac{1}{N}\sum_{i=1}^{N}\left(G_{i}^{0}-\bar{G^{0}}\right)\left(G_{i}^{1}-\bar{G^{1}}\right)}{\sigma_{0}\sigma_{1}}$$

where *N* is the number of data points in the conductance signatures, $G_{i}^{0}$ is the *i*-th conductance of the baseline signature with the average value at $\bar{G^{0}}$; $G_{i}^{1}$ is the *i*-th conductance of the signature at the curing age of interest whose average value is $\bar{G^{1}}$; *σ*_0_ and *σ*_1_ are the standard deviations of the baseline and the conductance signatures of interest, respectively.

**Figure 8:**
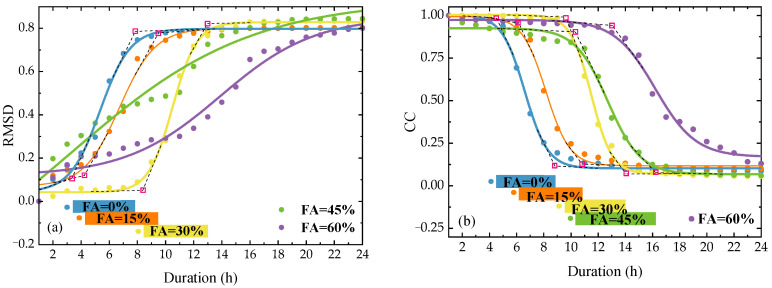
Statistical metrics (RMSD and CC) for SCC specimens with different FA replacement ratios during curing.

Figure 8a,b shows the changing curves of RMSD and CC with the hydration duration for SCC specimens with different FA replacement ratios. In each RMSD curve, the final setting can be easily observed as the fitted inflection when the RMSD converges at ~0.8, but the initial setting cannot be recognized from the fitted inflection. Whereas in each CC curve, both setting times can be identified by the two evident inflections. Before the first inflection, the CC decreases slowly from 1.0 to ~0.95, which indicates a strong correlation between the baseline signature and that of the SCC paste in the liquid state. Beyond the second inflection, the CC is nearly constant at ~0.06, indicating little correlation between the baseline signature and that of an SCC specimen in solid state. Between them, the CC decreases rapidly with curing, indicating a quick decrease in the correlation of the conductance signatures between liquid and solid states. It can be concluded that the CC performs better than the RMSD in indicating the initial and final settings.

As reported, the RMSD and CC can be utilized to quantify the variation and correlation of the conductance signatures recorded with curing, respectively. Although these statistical indices perform well in indicating the hydration states, some fitted inflections between consecutive states are not accurate enough to capture the state transition. For instance, the RMSD for each case is too sensitive to indicate the initial setting, as seen in Figure 8a, and the CC for the cases with high FA replacement ratios exhibits some uncertainties in the liquid state, as shown in Figure 8b. The transition duration of each changing curve is illustrated as a color bar and presented in Figure 8. It can be seen that both settings, indicated by RMSD or CC, increase with the increase in the FA replacement ratio. However, the transition duration elongation with the FA replacement ratio cannot be visually observed.

In the previous approach, the hydration state of SCC was correlated with predominant resonance shifts, a feature change in the conductance signature. In this approach, statistical metrics such as RMSD or CC reflect the overall changes in conductance signatures obtained in SCC with respect to a baseline. Both demonstrate the dynamic interaction between the PZT patch and its surrounding concrete. However, in the two-DOF model, the dynamic interaction reflects the combined effect of both the epoxy bonding layers and the host concrete. If the effect of the epoxy bonding layers cannot be neglected, it is difficult to identify the independent mechanical impedances of the host concrete. Thus, for quantitative identification of setting times, the effect of epoxy bonding layers should be extracted from the overall conductance signatures.

### 4.4. A PCA-Based Approach for Quantitative Hydration Monitoring in SCC

Principal component analysis (PCA) is an unsupervised machine learning method that can be used for dimensional reduction or feature extraction of the original high-dimensional data while retaining its original trend and shape. In PCA, multiple regression analyses are usually performed by projecting the original data onto lower dimensions called the principal component space. This would simplify the original correlated data into new uncorrelated data with lower dimensions [39]. Thus, by dimensionality reduction, key features of the original data may emerge on some reduced principal components with a minimized correlation.

In the hydration process, conductance signatures for each specimen were recorded every hour after casting. Each data can be expressed as {$F_{i}^{k}$, $G_{i}^{k}$}, where the two data sets (*F* and *G*) denote the respective frequency and amplitude of the conductance for the *i*-th specimen (*i* = 1, 2, …, n) at the *k*-th hour (*k* = 0, 1, 2, …, 24):

(8) $$F_{i}^{k}={\left[F_{1}^{k},F_{2}^{k},F_{3}^{k},\ldots ,F_{n}^{k}\right]}^{T}$$

(9) $$G_{i}^{k}={\left[G_{1}^{k},G_{2}^{k},G_{3}^{k},\ldots ,G_{n}^{k}\right]}^{T}$$

To eliminate the computational error caused by the dimensional difference between *F* and *G*, the raw data matrix {$F_{i}^{k}$, $G_{i}^{k}$} was standardized to obtain *Y_ij_* before the principal component analysis.

(10) $$Y=\left(\begin{array}{c} Y_{11},Y_{12},\ldots ,Y_{1i},\ldots ,Y_{1n}\\ Y_{21},Y_{22},\ldots ,Y_{2i},\ldots ,Y_{2n} \end{array}\right)$$

After the standardization, the vector of *Y_ij_* is rotated and expanded to obtain the R matrix. Each component in R is a real symmetric matrix composed of corresponding correlation coefficients.

(11) $$R=YY^{T}$$

(12) $$R=A\Lambda A^{T}$$

where A is an orthogonal matrix, and the k-th column of A (*α_k_*) is the *k*-th eigenvector of R. Λ is a diagonal matrix, and the *k*-th column of Λ (*λ_k_*) is the *k*-th eigenvalue of R. Each principal component can thus be converted into Z using the following formula:

(13) $$Z=A^{T}Y$$

The principal components are independent of each other. The contribution of each principal component decreases with the eigenvalue (*λ_k_*) and its corresponding unit eigenvector (*α_k_*) in the order of *k*. In this case, R is a two-dimensional matrix and has two eigenvalues, *λ*_1_ ≥ *λ*_2_ ≥ 0. The contribution ratio of the first principal component (CR1) can be expressed as:

(14) $$\mathrm{CR}1=\lambda_{1}/(\lambda_{1}+\lambda_{2})$$

which represents the maximum feature variance of the original data sets (***F*** and ***G***) that corresponds to the hydration state of SCC at the *k* time instant. Based on the CR1, the state transition points, such as the initial and final settings in each specimen, can be easily recognized by the fitted inflections of the CR1 after curing. Furthermore, the minor variance due to the predominant resonance and measurement noises can be reflected by the contribution ratio of the non-principal component (CR2).

**Figure 9:**
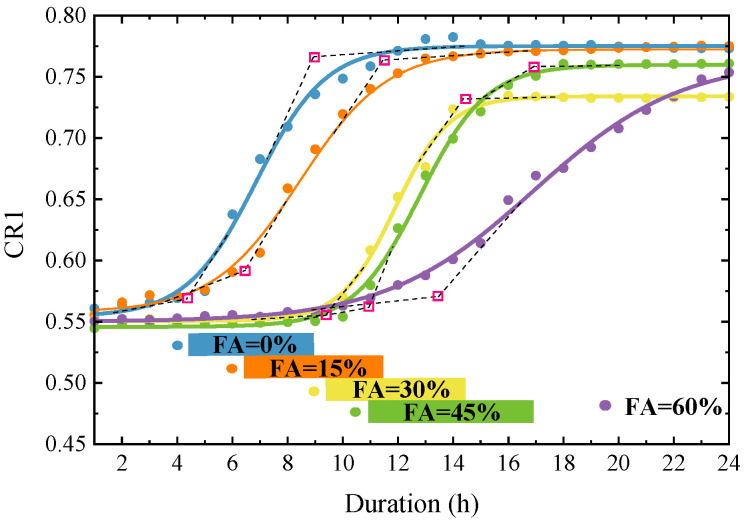
Variation of CR1 as a function of curing duration for SCC specimens with different FA replacement ratios.

Figure 9 shows the variation of CR1 as a function of curing time (in 24 h) for SCC specimens with different FA replacement ratios. For each specimen with a certain FA replacement ratio, the transition of the hydration states predicted by the CR1 curve is well consistent with that predicted by the other three approaches, i.e., the penetration resistance test, the resonance shifts, and the statistical metrics. Furthermore, the physical meaning of the CR1 can be explained by Equation (2), which describes the changes in the EMA of a two-DOF model. Before the initial setting, the dynamic stiffness of the SCC mixture (*K_s_*) is very small, and the modification coefficient on the mechanical impedance of host concrete (*ξ*) in Equation (2) is nearly 1.0. In this stage, the CR1s in cases with different FA contents are almost constant from the very beginning of the hydration. In the semi-solid stage, the *K_s_* increases rapidly alongside the liquid-solid state transition, and the *ξ* decreases accordingly, leading to the remarkable increase of the CR1. After the final setting, the *K_s_* becomes stable when the SCC mixture hardens and the CR1 finally converges at a certain value. This demonstrates that the changes in the CR1, theoretically determined by *K_s_* and *ξZ_s_*, can feature the hydration of the SCC at very early ages.

In the hardened stage, the convergent CR1 of each specimen varies with its FA replacement ratio. As shown in Table 4, the CR1 decreases from 0.766 to 0.753 as the replacement ratio increases from 0% to 30% as the replacement ratio increases from 0% to 30%, and then increases with the replacement ratio from 30% to 60%. Accordingly, an opposite changing trend can be found in the CR2 (CR2 = 1 − CR1). As demonstrated in Equation (2) of the two-DOF model, the CR2 consists of the contributions from the mechanical impedance of the embedded PZT sensor (*Z_a_*) and that from the interaction between the PZT sensor and surrounding concrete, which exhibits as the volume confinement acting on the PZT sensor. In this study, the maximum of CR2 occurs at a replacement ratio of 30%. This suggests a maximum stiffness ratio (*K_s_*/*K_b_*) in SCCs with a specific FA replacement ratio. It was reported that the SCCs might reach their corresponding largest strengths when the replacement ratios were within the range of 15–30% [40,41]. This range confirms the optimum FA replacement ratio (30%) with which the hardened concrete has the maximum stiffness ratio (*K_s_*/*K_b_*). This implies that, given a specific PZT mechanical impedance (*Z_a_*), the change in the equivalent stiffness of bonging layers (*K_b_*) with the replacement ratio is very small. The changes in CR2 with the FA replacement ratio, mainly reflecting the changes in the boundary constraints on the PZT sensors, are closely related to the shrinkage of the hardened concrete [42].

**Figure 10:**
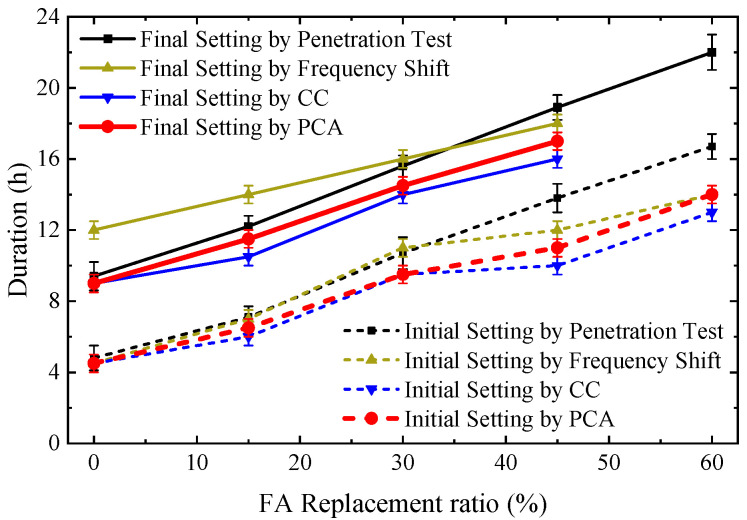
Comparison of the initial and final setting times for SCC specimens tested by different techniques.

Figure 10 shows the initial and final setting times predicted by different approaches for SCC specimens with different FA replacement ratios. Generally, all approaches predicted a similar increasing trend of the initial and final setting times with the increasing FA replacement ratios. This demonstrates that the hydration rate decreases with the increasing dosage of FA. The delayed settings were also reported by Yang et al. [5] via the penetration resistance tests in concrete with high FA contents. Furthermore, the duration elongation of the liquid-solid transition with FA content can be visually observed in the results predicted by PCA approach, as shown in Figure 10 and Table 5. The transition duration elongation predicted by PCA approach is 1.5 h in SCCs with FA replacement ratios increasing from 0% to 45%, which is comparable to the reported duration elongation (2 h) in SCCs with FA replacement ratios increasing from 0% to 40% [8,43]. For comparison, the transition durations for SCC specimens using different approaches are listed in Table 5. It can be noted that, among these approaches, the PCA-based one performs best in capturing the duration elongation between the initial and final settings without requiring a baseline. These advantages may render the PCA-based approach a robust and quantitative tool for concrete hydration monitoring under in-situ conditions.

## 5. Conclusions

This work studied the hydration process of self-compacting concrete (SCC) at very early ages based on the penetration test and EMI techniques. Based on the EMI tests, a novel PCA-based approach was proposed for quantitative hydration monitoring in SCC. The setting times predicted by this approach were compared with those obtained by the penetration tests and those predicted by resonance shifts and statistical metrics. Some conclusions are listed as follows:

(1) The initial and final setting times predicted by the statistical metrics and the CR1 are well consistent with those predicted by the penetration resistance test. However, using embedded PZT sensors for in-situ hydration monitoring is a further advantage of the EMI-based approaches.
(2) Similar three-stage hydration processes of fresh SCC mixtures can be identified by the resonance shifts, the statistical metrics, and the CR1 based on the measured conductance signatures. All three EMI-based approaches can predict the retardation of setting times with FA replacement ratio in SCC specimens, but only the PCA approach can accurately estimate the duration elongation of the liquid-solid transition with FA replacement ratio during curing.
(3) In the PCA approach, the CR1 can explicitly capture the dynamic stiffness changes in SCC specimens with curing duration, while the CR2 can implicitly reflect the boundary constraints on the PZT sensor induced by the shrinkage of the hardened SCCs. This approach can separate the mechanical impedance of the epoxy-packed PZT sensor from its host concrete, thus providing a robust method to monitor the very early-age hydration of SCC without requiring a baseline.

## Figures and Tables

**Table 1:** Electrical resistances and capacitances of PZT sensors before and after packing.

PZT Sensor Specimens	Electrical Resistance		Electrical Capacitance		
	Before Packing	After Packing	Before Packing	After Packing	Reduction
H1	∞	∞	2.29	2.10	8.30%
H2	∞	∞	2.22	2.03	8.56%
H3	∞	∞	2.31	2.12	8.23%
H4	∞	∞	2.35	2.01	14.47%
H5	∞	∞	2.33	2.00	14.16%

**Table 2:** Mix proportions for the test SCC specimens.

Specimens	Crushed Stone (kg/m^3^)	Sand (kg/m^3^)	Cement (kg/m^3^)	Fly Ash (kg/m^3^)	Limestone (kg/m^3^)	Water (kg/m^3^)	Water Reducing Agent (kg/m^3^)	Compressive Strength (MPa)
SCC-F0	784.0	756.9	497.7	0	21.7	191.5	5.2	39.14
SCC-F15	784.0	756.9	419.1	77.6	21.7	191.5	5.2	36.04
SCC-F30	784.0	756.9	341.5	155.2	21.7	191.5	5.2	42.12
SCC-F45	784.0	756.9	263.9	232.9	21.7	191.5	5.2	28.03
SCC-F60	784.0	756.9	186.2	310.5	21.7	191.5	5.2	22.14

**Table 3:** Setting times for SCC specimens with different FA replacement ratios predicted by penetration resistance tests.

Replacement Ratio of FA to Binder	Initial Setting Time (h)	Final Setting Time (h)	Duration (h)
0%	4.8	9.4	4.6
15%	7.1	12.2	5.1
30%	10.7	15.6	4.9
45%	13.8	18.9	5.1
60%	16.7	22.0	5.3

**Table 4:** CR1 calculated at initial and final settings for SCC specimens with different FA replacement ratios.

Specimens	SCC-F0	SCC-F15	SCC-F30	SCC-F45	SCC-F60
Initial Setting	0.570	0.590	0.555	0.560	0.570
Final Setting	0.766	0.764	0.753	0.759	-

**Table 5:** Time gaps between the initial and final settings by different approaches.

Specimen	Penetration Resistance Test (h)	Frequency Shift (h)	Amplitude Shift (h)	RMSD (h)	CC (h)	PCA (h)
SCC-F0	4.6	7.5	3.5	4.5	4.5	4.5
SCC-F15	5.1	7.0	5.0	5.5	4.5	5.0
SCC-F30	4.9	5.0	4.5	5.0	4.5	5.0
SCC-F45	5.1	7.0	5.5	-	6.0	6.0
SCC-F60	5.3	-	-	-	-	-

## Data Availability

Some data used during the study are available from the corresponding author by request.
